# Gene Expression Profiling in Coeliac Disease Confirmed the Key Role of the Immune System and Revealed a Molecular Overlap with Non-Celiac Gluten Sensitivity

**DOI:** 10.3390/ijms24097769

**Published:** 2023-04-24

**Authors:** Michele Sallese, Konstantinos Efthymakis, Michele Marchioni, Benedetto Neri, Beatrice Dufrusine, Enrico Dainese, Marta Di Nicola, Matteo Neri

**Affiliations:** 1 Department of Innovative Technologies in Medicine and Dentistry, University “G. d’Annunzio” of Chieti-Pescara, 66100 Chieti, Italy; 2 Center for Advanced Studies and Technology (CAST), ‘G. d’Annunzio’ University of Chieti-Pescara, 66100 Chieti, Italy; efkn78@gmail.com (K.E.); bdufrusine@unite.it (B.D.); 3 Department of Medicine and Ageing Sciences, ‘G. d’Annunzio’ University of Chieti–Pescara, 66100 Chieti, Italy; 4 Laboratory of Biostatistics, Department of Medical, Oral and Biotechnological Sciences, ‘G. d’Annunzio’ University of Chieti–Pescara, 66100 Chieti, Italy; michele.marchioni@unich.it (M.M.); marta.dinicola@unich.it (M.D.N.); 5 Gastroenterology Unit, Department of Systems Medicine, University ‘Tor Vergata’ of Rome, 00133 Roma, Italy; benedettoneri@gmail.com; 6 Department of Bioscience and Technology for Food Agriculture and Environment, University of Teramo, 64100 Teramo, Italy; edainese@unite.it

**Keywords:** celiac disease, gene expression profiling, intestinal biopsies, non-celiac gluten sensitivity

## Abstract

Coeliac disease (CeD) is an immune-mediated disorder triggered by the ingestion of gluten and an as yet unidentified environmental factor in genetically predisposed individuals. The disease involves a major autoimmune component that primarily damages the intestinal mucosa; although, it also has systemic involvement. The Th1 inflammatory response is one of the main events leading to mucosal damage; although, enterocytes and the innate immune response also participate in the pathological mechanism. In this study, we performed an analysis of the gene expression profile of the intestinal mucosa of patients with active disease and compared it with that of patients who do not suffer from gluten-related disorders but report dyspeptic symptoms. This analysis identified 1781 differentially expressed (DE) genes, of which 872 were downregulated and 909 upregulated. Gene Ontology and pathway analysis indicated that the innate and adaptive immune response, in particular the Th1 pathway, are important pathogenetic mechanisms of CeD, while the key cytokines are IL27, IL21, IL2, IL1b, TNF, CSF2 and IL7, as well as type I (IFNA1, IFNA2) and type II (IFNG) interferons. Finally, the comparison between the DE genes identified in this study and those identified in our previous study in the intestinal mucosa of patients with non-celiac gluten sensitivity (NCGS) revealed a high degree of molecular overlap. About 30% of the genes dysregulated in NCGS, most of which are long non-coding RNAs, are also altered in CeD suggesting that these diseases may have a common root (dysregulated long non-coding RNAs) from which they develop towards an inflammatory phenotype of variable degree in the case of CeD and NCGS respectively.

## 1. Introduction

Coeliac disease (CeD) is an immune-mediated enteropathy that can occur in genetically predisposed individuals due to the expression of HLA DQ2 or DQ8 [1,2,3]. Triggers for CeD are gluten ingestion as well as less defined events that may include intestinal viral infections, intestinal dysbiosis or stressful events. The pathogenetic mechanism is rather complex and involves the adaptive and possibly the innate immune response, as well as the contribution of enterocytes and myofibroblasts. In fact, several classes of lymphocytes are recruited in the coeliac mucosa, in particular γ/δ and α/β CD8^+^ T-lymphocytes increase within the intestinal epithelium (intraepithelial lymphocyte, IEL), while CD4^+^ T-lymphocytes α/β, eosinophils, neutrophils and mast cells increase in the lamina propria [3,4,5,6,7].

Gluten-derived peptides are highly affine for HLA DQ2 and DQ8 and deamidation via transglutaminase 2 (TG2) further increases this affinity [8]. Thus, efficient gluten peptide presentation activates several T cell subtypes that will result in the production of cytokines including interferon-γ (IFNG) and interleukin (IL) 17 [9,10,11]. Moreover, IL15 and IFNA involvement suggests a role of the innate immune response in CeD [9,12,13]. The interaction of gluten with enterocytes induces the release of tumour necrosis factor TNFα, IL8 and IL25 as well as the expression of the stress molecules major histocompatibility complex class I chain-related molecule A (MICA) and HLA-E, which can be targeted by cytotoxic T lymphocytes and induce epithelial cell damage [9,14]. The immune response also generates auto-antibodies against TG2 and gliadin, one of the gluten proteins. The measurement of anti-TG2 IgA by ELISA and its reactivity to endomysium (immunofluorescence) are used to diagnose CeD [15]. In addition, anti-deamidated gliadin antibodies can also be monitored in the diagnosis of CeD. As CeD patients are sometimes deficient in IgA, IgG against TG2 is measured as an alternative [15].

The development of inflammation leads to profound remodelling of the intestinal mucosa, characterised by atrophy of the villi and hyperplasia of the crypts [16]. These alterations are classified according to the Marsh–Oberhuber scale, where grade I indicates an intact morphology with an IEL infiltrate, grade II shows proliferation of the crypts in addition to an IEL infiltrate, while grade III presents atrophy of the villi, hyperplasia of the crypts and swelling, whereby the mucosa appears flattened [17]. Histological analysis of intestinal mucosa biopsies is the gold standard to diagnose CeD in adults with positive serology [4]. Some aspects of CeD pathology are well characterised, while others, such as the contribution of innate immunity or that of non-immune cells (enterocytes and myofibroblasts), are less well studied.

In some cases, apart from the symptoms, the ingestion of gluten does not trigger the characteristic CeD serology nor the alteration of the intestinal mucosa, so these patients cannot be considered coeliac. However, if they test positive for the Salerno’s expert criteria, they are considered to have non-celiac gluten sensitivity (NCGS) [18,19]. The Salerno expert’s criteria are a complex protocol in which, basically, patients test positive for NCGS if they report a reduction in or exacerbation of symptom severity by at least 30% when given a GFD or a gluten-containing diet [19].

The molecular mechanisms of NCGS are still poorly understood; although, the slight increase in intestinal intraepithelial lymphocytes (IEL) and the production of cytokines in situ suggest the important role of the immune response [20]. Specifically, it was reported that NCGS intestinal mucosa showed an upregulation of toll-like receptor 2 (TLR2), whereas IL6 and IL21 were not significantly affected. A possible involvement of CXCL10 and its receptor CXCR3 has also been reported [21,22]. Furthermore, unlike CeD, intestinal permeability in NCGS was reduced and claudin-4 (CLDN4), a key protein of epithelial cell tight-junctions, was upregulated. Until now, it had been assumed that the intestinal mucosa was rather preserved in NCGS apart from the increased IEL [23]. In contrast, a very recent and thorough multicentre study reported that NCGS subjects show a reduction in villus height and the ratio of villus height to crypt depth compared to control subjects; although, the alterations are less pronounced than those found in CeDs [16].

Functional dyspepsia (FD), defined by the Rome IV criteria, is a common clinical condition characterised by upper gastrointestinal symptoms, abdominal pain and bloating, postprandial fullness and nausea that do not depend on any organic alteration. FD tends to cluster in families, suggesting the role of genetic factors in the occurrence of this condition. Over time, several studies have pointed at gene polymorphisms modulating disturbances in gastroduodenal sensory and motor functions. However, as analysed in a recent systematic review [24], no consistent association was found between most of the reported gene polymorphisms and FD. Moreover, a recent gene expression profiling study, conducted on duodenal biopsies of children, identified three candidate genes associated with FD pain [25]. Given the lack of major measurable molecular changes in FD, these patients represented a good control for our study.

In the present research, we decided to analyse gene expression changes in the intestinal mucosa of coeliac patients at the time of diagnosis, with the aim of improving our knowledge of CeD pathology. Intestinal biopsies of controls and CeD patients showed dissimilar gene expression profiles. Gene ontology analysis confirmed that T-lymphocyte activation is the main pathogenic event in CeD. In addition, a significant increase in cell proliferation was observed, supporting the idea that the shortening of the villi results mainly from crypt overgrowth. Protein–protein interactions analysis has revealed many other small networks suggesting that other biological functions are involved in CeD pathology. In conclusion, our results indicate that the innate and adaptive inflammatory immune response is an important pathogenetic mechanism of CeD and that IL27, IL21, IL2, IL1β, TNF, colony-stimulating factor 2 (CSF2) and IL7, as well as type I (IFNA1, IFNA2) and type II (IFNG) interferons, are the soluble factors orchestrating this inflammatory response. Interestingly, part of the DE genes in CeD overlaps with DE genes identified in our previous study on NCGS [26], indicating that these gluten-related disorders share parts of the pathogenetic mechanism, in agreement with the study by Rostami et al. [16].

## 2. Results

### 2.1. Gene Expression Changes in Intestinal Biopsies of Coeliac Patients

**Figure 1:**
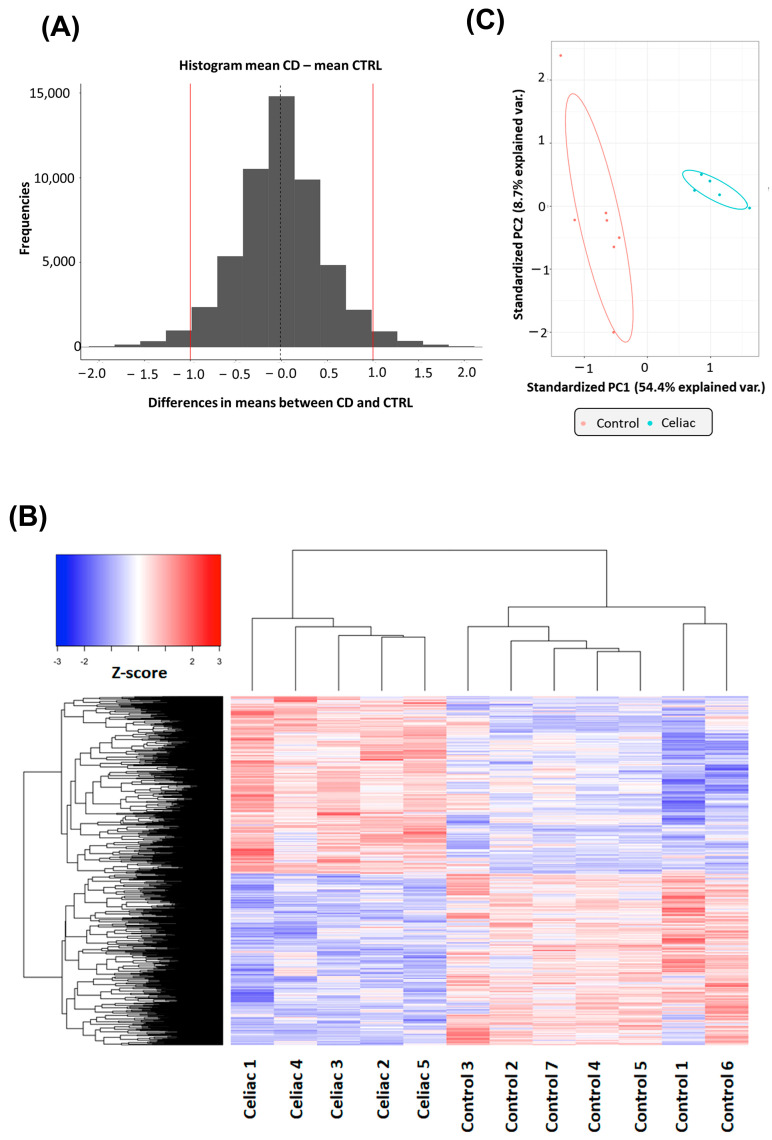
Comparison of gene expression levels in the intestinal mucosa of CeDs and controls: (**A**) frequencies of gene expression levels differences between CeDs and controls. (**B**) Heat map of the modulated genes. For the heatmap, a Z-score normalization is performed on the normalized read counts across samples for each gene. Z-scores are computed on a gene-by-gene (row-by-row) basis by subtracting the mean and then dividing by the standard deviation. The computed Z score is then used to plot the heatmap. (**C**) Principal component (PC) analysis of the DE transcripts. A scatter plot of the first two components (PC1 and PC2) of the DE-expressed transcripts between CeD and controls with 95% confidence ellipses is shown.

To assess the gene expression profile in CeD, we recruited five newly diagnosed patients who were on a free diet and had severely damaged intestinal mucosa, with a Marsh grade in most cases above 2 (Table 1). Patients’ characteristics are described in the ‘Methods’ section. As controls, we enrolled seven patients with gluten-independent dyspeptic symptoms who required endoscopic examination. Total RNA extracted from duodenal biopsies of CeD and controls was used to measure gene expression by microarray. Probes that revealed an expression difference of less than one were filtered out, while the remaining 3061 were selected for statistical analysis (Figure 1A). T-testing followed by FDR correction (FDR ≤ 0.05) identified 1877 probes targeting differentially expressed (DE) genes. Since 96 transcripts were recognized by multiple probes, we have indeed 1781 DE genes, 872 of which were downregulated and 909 were upregulated (Supplementary Table S1). A heat map showing the levels of variation in DE genes is depicted in Figure 1B. The 1781 DE genes included 328 as yet unclassified transcripts, 591 non-coding RNAs (e.g., antisense, lnc, LINC, LOC, SNORA, SNORD and pseudogenes) and 862 protein-coding transcripts (Supplementary Table S1). This means that, excluding unclassified transcripts, about 60% of the remaining DE genes encode for proteins. A principal component analysis, performed to reduce the dimensionality of the data set, showed that 54% of the variance can be explained by the first component and is associated with CeD status, while a further 8.7% is explained by the second component, which is not associated with CeD (Figure 1C). Note that the principal components variables are sufficient to distinguish CeD from controls (Figure 1C). Next, the least absolute shrinkage and selection operator (LASSO) penalized logistic regression, used to identify transcripts that may act as biomarkers predictive of CeD diagnosis, identified the potassium channel KCNQ2 and the antimicrobial cationic protein AZU1. The Receiver operating characteristic (ROC) analysis confirmed that KCNQ2 and AZU1 genes are sufficient to distinguish CeD from controls with an area under the curve (AUC) approaching 1.

### 2.2. Functional Analysis of DE Gene in Intestinal Biopsies of Coeliac Patients

**Figure 2:**
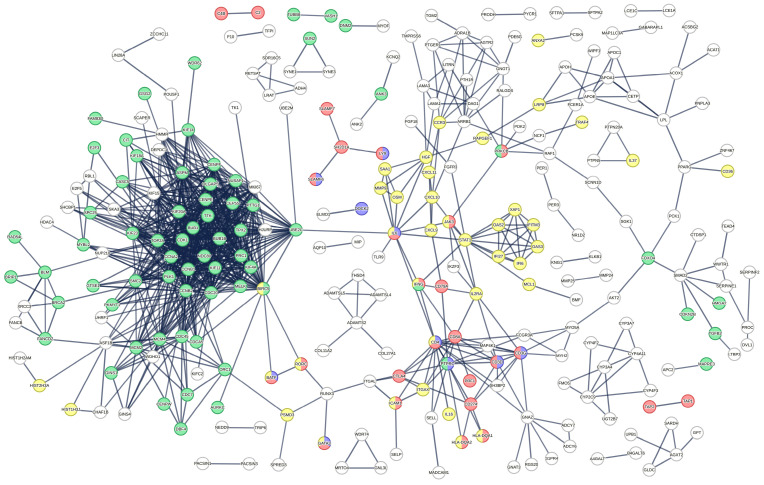
Protein–protein interaction network based on the DE genes. DE gene network based on the STRING database with confidence level set to 0.9. Genes involved in the cell cycle process are depicted by green nodes. Genes involved in the adaptive immune response are depicted by the red nodes. Genes involved in T-cell selection are depicted by the blue nodes. Genes involved in the cytokine-mediated signalling pathway are depicted by the yellow nodes. The rest of the functionally interconnected DE genes are represented by empty nodes.

In order to assess the reliability of our findings, we searched for possible relationships between DE genes using the STRING protein–protein interaction (PPI) database [27]. Note that of the 1781 DE transcripts, only 860 protein-coding transcripts were present in the STRING database. Using a standard STRING stringency (0.4), these 860 genes (nodes) are expected to participate in 2319 random interactions (edges), whereas our data set produced 4449 interactions (*p*-value of PPI enrichment < 1.0 × 10^−16^, average node rank 10.3 and average local clustering coefficient 0.407), underlining the reliability of our data. Using the highest stringency (0.9), the number of random edges is expected to be 308 while our data set presents 930 interactions among the nodes with an average node degree of 2.16 and an average local clustering coefficient of 0.236 (PPI enrichment *p*-value: <1.0 × 10^−16^). These analyses support the concept that DE-expressed genes are not picked by chance but rather belong to altered cellular functions. The PPI network generated by DE transcripts shows several clusters of highly connected nodes, indicative of altered functions rather than isolated dysregulated genes (Figure 2). The cluster of genes involved in the cell cycle process is in green, the one involved in the adaptive immune response is in red, the one involved in T-cell selection is in blue and the one involved in the cytokine-mediated signalling pathway is in yellow (Figure 2).

We then evaluated the enrichment of gene ontologies (GO) in the data set to obtain information on the functions of the clustered nodes, as they are potentially involved in CeD. According to the GO analysis, the biological process involved in CeD includes T-helper 17 cell differentiation, T cell selection, adaptive immune response, cytoskeleton organization, regulation of cell adhesion, programmed cell death, response to stress and several processes related to the cell cycle. Analysing the enrichment of pathways in the KEGG, Reactome and wiki databases, we obtained results similar to those obtained by GO plus the involvement of the LDL, HDL and TG metabolic pathways. The complete lists of statistically enriched biological processes and pathways can be found in Supplementary Tables S2 and S3. Finally, the enriched molecular functions associated with DE genes are cytoskeletal protein binding, microtubule binding and tubulin binding.

### 2.3. Ingenuity Pathway Analysis of DE Genes

To further investigate the functional significance of dysregulated gene expression in CeD, we performed Ingenuity Pathway Analysis (IPA). First, we focused on ‘canonical pathways’, i.e., well-established and manually curated metabolic and signalling pathways.

DE genes were significantly enriched in different canonical pathways including the macrophage classical activation signalling pathway, Th1 pathway and interferon signalling (Supplementary Table S4). This result indicates a central role of both the innate and adaptive immune response in CeD. A more extensive list of significantly activated canonical pathways (Z-score > 2), based on the direction of expression changes, is shown in Supplementary Table S4. Among the inhibited canonical pathways was that of IL10 signalling (−log *p*-value 1.37; z-score −2.111), which is however consistent with an activated inflammatory response condition.

We then performed an ‘Upstream Analysis’ to identify key molecules capable of inducing the observed changes in gene expression. Upstream molecules whose activation could generate such changes in gene expression include IFNG (z-score 4.24, *p*-value 1.77 × 10^−8^), immunoglobulins (z-score 4.501, *p*-value 1.64 × 10^−7^), TNF (z-score 4.9, *p*-value 4.37 × 10^−9^), CSF2 (z-score 5.354, *p*-value 2.68 × 10^−16^) and Eldr (z-score 5.657, *p*-value 5.32 × 10^−31^). A more extended list of potential activators is reported in Supplementary Table S5. The first three genes are classical regulators of the immune response, while Eldr is a recently identified long non-coding RNA that controls gene expression. The genes identified in this study and potentially controlled by Eldr are shown in Supplementary Figure S1. In addition, the activities of upstream regulators, interleukin-1 receptor antagonist (IL1RN; z-score −3.228, *p*-value 4.73 × 10^−4^) and the immune-inhibitory receptor CTLA4 (z-score −3.056, *p*-value 2.64 × 10^−7^), are repressed.

We next evaluated the diseases and physiological functions that can be inferred from the DE gene repertoire identified in this study. Using the IPA module ‘Disease and Functions’, we found that DE genes are predictive of the development (z-score 3.507, *p*-value 1.07 × 10^−6^), homeostasis (z-score 3.357, *p*-value 2.72 × 10^−7^), migration (z-score 2. 92, *p*-value 1.26 × 10^−7^) and binding (z-score 2.517, *p*-value 2.67 × 10^−7^) of T-cells, as well as of the migration of mononuclear leukocytes (z-score 2.899, *p*-value 9.14 × 10^−10^) and lymphocytes (z-score 2.81, *p*-value 2.84 × 10^−9^). A more extended list of diseases and functions can be found in Supplementary Table S6.

**Figure 3:**
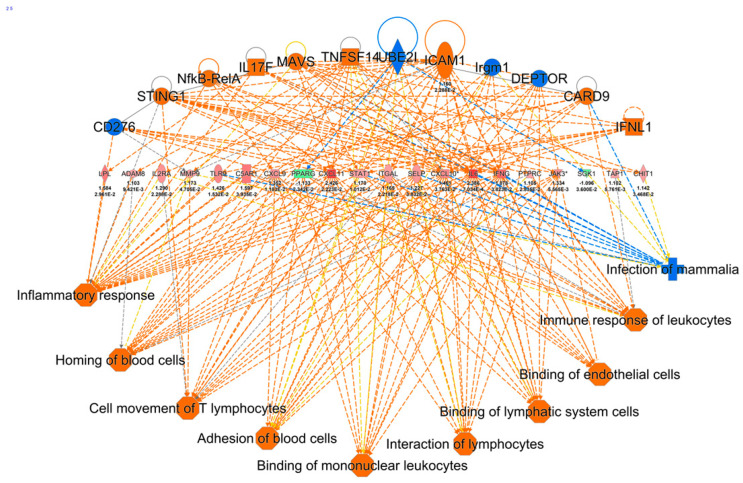
Gene network linking the upstream regulator to the downstream phenotype by means of DE genes.

We also evaluated the functional networks linking the upstream regulators with specific downstream phenotypes by means of the genes in our data set. This analysis was performed by the IPA function called ‘Regulator Effects’. The reliability of a network is assessed by a consistency score that measures the causal consistency of the inferred network and the density of connections. Downstream phenotypes with the highest consistency scores include the adhesion of blood cells, binding of lymphatic system cells, binding of mononuclear leukocytes, cell movement of T lymphocytes, homing of blood cells, immune response of leukocytes, and inflammatory response. These phenotypes can be modelled by twelve regulators, belonging to the families of adhesion molecules, cytokines and transcription factors, by means of twenty target proteins, mainly cytokines (Figure 3).

**Figure 4:**
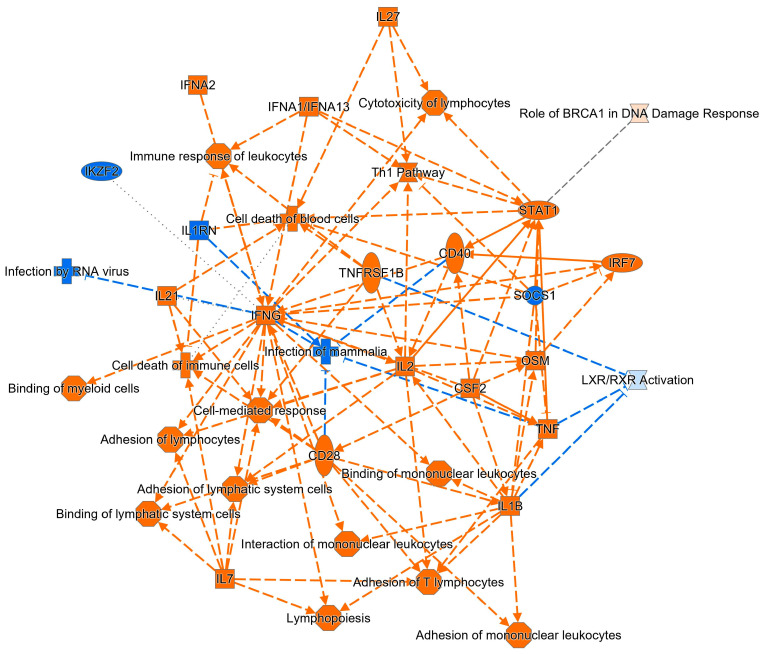
Graphical summary of the key molecules and functions involved in CeD.

Finally, a graphic summary of our findings shows the key molecules and cellular functions involved in CeD. In the scheme, it is possible to appreciate the role of key cytokines including IL27, IL21, IL2, IL1b, TNF, CSF2 and IL7 as well as interferon type I (IFNA1, IFNA2) and II (IFNG) on innate and adaptive immune response including the Th1 pathway (Figure 4).

### 2.4. Comparative Analysis of DE Genes in Celiac Disease and NCGS

In our previous study, we analysed the gene expression profiles of the intestinal mucosa of NCGS patients and compared them with controls, which resulted in the identification of 300 DE transcripts [26]. CeD and NCGS are both triggered by gluten ingestion and present mostly overlapping clinical symptoms; however, it is not known whether they also share some of the pathogenetic mechanisms. To provide some insight into this topic we examined whether the DE genes identified in this study were the same as those identified in our previous study on NCGS patients [26].

Interestingly, 83 DE transcripts are shared between CeD and NCGS and account for about 30% of DE transcripts previously identified in NCGS (Supplementary Table S7). We would like to emphasise the fact that the controls were exactly the same subjects in both studies (this one and [26]).

Unfortunately, it was difficult to understand the functional significance of this overlap because 23 of these transcripts are completely unknown, 37 are uncharacterised or poorly characterised non-coding RNAs and only 23 are protein-coding transcripts (Supplementary Table S7). Based on STRING analysis, these 23 genes are mostly independent and only 5 of them constitute a small functional cluster involved in the control of circadian rhythm. IPA analysis of upstream regulators deduced that the DE genes common to CeD and NCGS can be controlled by miRNAs including miR-6887-3p (z-score 2.449, *p*-value 1.13 × 10^−2^), miR-802-3 (z-score 2.236, *p*-value 2.10 × 10^−3^), miR-7108-3p (z-score 2.236, *p*-value 2.30 × 10^−3^) and miR-329-3p (z-score 2.219, *p*-value 2.30 × 10^−2^). The genes potentially regulated by each of these miRNAs are shown in Supplementary Table S8.

## 3. Discussion

In the present study, we analysed the gene expression profile of duodenal biopsies from patients with newly diagnosed CeD. To increase reproducibility and reduce interpersonal variability, only patients with a high degree of intestinal damage were recruited. Patients who had never reported gluten-related symptoms, but who were eligible for gastroduodenoscopy because they reported dyspeptic symptoms, were recruited as controls.

Total RNA isolated from duodenal biopsies was used for microarray analysis. Comparison of gene expression levels in CeDs versus controls led to the identification of 1781 DE genes, almost equally distributed between upregulated and downregulated. Excluding the uncharacterized genes, about 60% of the known DE genes were protein-coding transcripts. In contrast, a similar analysis in NCGS revealed that only 35% of DE genes encode for proteins [26]. Using PCA, we demonstrated that DE genes can be used as a biomarker for CeD. Interestingly, even measuring the expression of a single gene such as AZU1 or KCNQ2 is sufficient to identify patients affected by CeD. AZU1 is a protein-coding gene present in azurophil granules of neutrophils and is involved in innate immune response [28]. Probably, this gene is so relevant because the neutrophils themselves are; in fact, the neutrophil/lymphocyte ratio can be used as a sensitive marker for the diagnosis of CeD [29]. On the other hand, it is more difficult to speculate on the role of KCNQ2, which is mainly expressed in neurons and involved in epilepsy [30]. Neurological manifestations are part of the spectrum of extraintestinal components of CeD [31] and individuals with CeD are at increased risk of epilepsy, which may affect approximately 5% of these patients, as demonstrated in previous studies [32]. Epilepsy has been associated with occipital calcifications, but other factors such as malabsorption of vitamin B12, local deposition of TG2 antibodies leading to impaired function of immune complexes, and vasculitis have been described [33]. Further study might assess the relevance of the association of genetic factors, as described in this paper, with the occurrence of epilepsy.

Analysis of protein–protein interactions of DE genes revealed a highly interconnected network of proteins with clear clusters involved in the mitotic cell cycle process and stimulus response, suggesting that immune cells are reacting to an external stimulus. Probably, we observed a positive regulation of the cell cycle because of immune cell proliferation and mucosa regeneration. This analysis also suggested a metabolic dysregulation of lipids including both cholesterol and triglycerides. Indeed, lower levels of cholesterol have been reported in CeD patients at diagnosis [34].

A more in-depth analysis conducted on the IPA platform revealed the activation of the innate and adaptive inflammatory response. As previously reported immunoglobulins, interferons and TNF appear to be the key molecules orchestrating the immune response in CeD [2,35]. In addition, we identified the long non-coding RNA named Eldr, which has been recently shown to be important in tumour proliferation, specifically in the control of the cell cycle [36]. Eldr could be a key player in the activation of the mitotic cell cycle process we observed. Another potentially important upstream regulator is the Interleukin-1 receptor antagonist, which is possibly downregulated to create a permissive environment for the development of the inflammatory response. The importance of IL1RN is underlined by the occurrence of autoinflammatory disorders in IL1RN-deficient patients [37].

Surprisingly, CTL4, a T-cell activation inhibitory receptor was predicted to be downregulated based on the overall transcriptomic changes we observed, but was actually upregulated in our CeD samples. The upregulation of CTLA4 on intestinal mucosa has been reported previously [38]. Further studies are needed to address these somewhat inconsistent results and clarify the important role of CTLA4 in CeD.

Based on the main physiological functions we identified in the biopsies of CeD patients, we deduce that T-cell development, migration and binding are crucial in this disease. Note that our gene expression analysis was performed on duodenal biopsies, yet we identified changes associated with immune response activation rather than enterocytes. This underlines the sensitivity of our approach, which was able to detect alterations associated with a minority of cells infiltrated in whole tissue. The centrality of immune activation versus the role of enterocytes in CeD was also supported by our analysis of functional networks, which revealed extensive involvement of most immune cells and classical mediators of inflammation.

The key cytokines that emerged in this study include IL27, IL21, IL2, IL1β, TNF, CSF2 and IL7 and interferons alpha and beta. IL27 is released by dendritic cells and macrophages and has pleiotropic effects on the innate immune response by acting on natural killer cells, granulocytes, and dendritic cells (inhibitory) and macrophages themselves [39]. IL27 acts on naïve CD4^+^ T cells to generate Th1 cells while inhibiting Th2 and Th17 differentiation [39]. IL27 is upregulated in CeD and supports the production of IL21 and IFNG, two important players in this disease [40,41]. A previous study associated IL27 with different autoimmune diseases [42]. IL21, in analogy to IL27, is a cytokine acting at the crossroad between innate and adaptive immune response. IL21 is produced by multiple immune cells, especially activated T cells, and regulates the differentiation and proliferation of several immune cells (macrophages, natural killer cells, B cells and cytotoxic T cells) [43]. IL21 has been involved in lupus and psoriasis autoimmune diseases [44]. In addition, celiac patients present elevated serum levels of IL21 that correlate with mucosal damage and anti-TG2 IgA autoantibodies [45]. IL2 is secreted by T-cells and promotes the proliferation of T- and B-lymphocytes. In fact, coeliac patients show an increase in circulating IL2 levels that correlates with disease activity/severity [46]. IL1β is a pro-inflammatory cytokine mainly secreted by innate immune cells and promotes T- and B-cell activation. IL1β levels are increased in celiac disease [47], and together with TNF and IFNG, it favours intestinal permeability, a characteristic feature of celiac disease [48]. TNF, analogous to IL1β, is a pro-inflammatory cytokine produced by macrophages and T-cells and contributes to tissue damage in CeD. Studies have shown that TNF levels are elevated in the small intestine of subjects with active disease [35,49]. CSF2 is a cytokine that regulates the production and functions of granulocytes macrophages. A recent study reported elevated levels of CSF in CeD [41]. IL7 is primarily produced by epithelial cells in the intestine and regulates the development of T- and B-cells. Although high levels of IL7 have been reported, its role in CeD is not well established [50]. According to a recent study, this cytokine could support the survival and activation of IEL [50]. Interferons type I produced by macrophages and dendritic cells contribute to the Th1 response in CeD [51]. Finally, as suggested by the multiple connections shown in Figure 4, IFNG plays a central role in CeD, as its levels generally increase many-fold in these patients and are strongly correlated with intestinal damage and remodelling [52].

The conclusions of this study must be taken with some caution as they are based on a limited number of patients. We recognise this limitation and have therefore made every technical and statistical effort to prove the reliability of our results. Finally, we believe that, despite the low number of patients, our results are also reliable because they identified the molecular alterations previously reported in CeD.

Eventually, we examined the molecular overlap between CeD and NCGS. These diseases present similar symptoms in response to gluten ingestion, but neither the morphological alteration of the intestinal mucosa nor the serological markers of CeD are observed in patients with NCGS [53]. To date, the cause of NCGS as well as its pathological mechanism is still unknown. Our study indicates that NCGS shares about 30% of its molecular alterations with CeD. This suggests that although CeD and NCGS are fundamentally different diseases, some molecular features of their pathogenetic mechanism are shared. In particular, non-coding regulatory RNAs, which represent the main group of dysregulated genes in NCGS and account for approximately 60% of shared DE transcripts, could also be important in CeD pathology. The reason why these molecular overlaps have not been identified before is possibly due to the fact that non-coding RNAs are still poorly studied.

## 4. Materials and Methods

### 4.1. Patients Participating in the Study

Symptomatic patients with a diagnosis of CeD made at the gastroenterology unit of the SS Annunziata Hospital in Chieti were considered for enrolment in this study. In accordance with international standards, the diagnosis of CeD was established in patients with positive serological tests for anti-TGA and anti-EMA and altered morphology of four duodenal biopsies. Those with intestinal grade II lesions or higher, according to the Marsh scale, were enrolled in this study. Note that the CeD patients were all HLA DQ2 and/or DQ8 positive; their complete characteristics are shown in Table 1. Ethical approval was obtained from the Clinical Research Ethics Committee dell’Università degli Studi “G. D’Annunzio” and Azienda Sanitaria Locale (ASL) N2 Lanciano-Vasto-Chieti; report number 13, 18 July 2013. Furthermore, written informed consent was obtained from all the participants. The biopsies used in this study were taken in proximity to those used for diagnostic purposes. As a control, we used biopsies from patients who were prescribed endoscopic examination because reported dyspeptic symptoms were defined according to the Rome III criteria. Note that these very controls were used in our previous comparative study of gene expression in NCGS [26].

### 4.2. RNA Extraction, Microarray Analysis, Statistical Testing and Functional Analysis of DE Genes

Total RNA extraction, microarray analysis, statistical testing and functional analysis were performed as described in our previous study [26,54]. Briefly, total RNA was extracted from duodenal biopsies of seven controls and five CeD using the RNAsy extraction kit from Qiagen (Milan, Italy) according to the manufacturer’s instructions. Microarray analysis was performed by outsourcing to “Consorzio Futuro in Ricerca” Ferrara, Italy, using Agilent Technologies. Data transformation was applied to set all the negative raw values at 1.0, followed by quantile normalization and log2 transformation.

The values of continuous variables, such as gene expression, were checked for normal distribution using the Shapiro–Wilk test and given as mean and standard deviation. Qualitative data were summarized as frequencies and percentages (SD). Each group’s results were presented separately. To assess the variations in quantitative and qualitative features between groups, the Mann–Whitney U test and Pearson’s chi-squared test were used, respectively.

Transcripts with values greater than 1 were chosen as differentially expressed (DE) transcripts by calculating the absolute difference between the mean expression in celiac patients and controls. Using the FDR cutoff of 0.05, the Student’s *t*-test was used to choose transcripts where there was a statistically significant difference between the mean expression of celiac patients and controls [55].

Unsupervised principal component analysis (PCA) was used to decrease the dimensionality of a microarray data set into two components, principal component (PC) 1 and PC2. Using the R function “prcomp”, PCA was performed as an “unsupervised” analysis to define the variance between microarray data from two groups. To establish the percentage of variation explained by each PC, the proportion of variance was also determined.

To find DE transcripts indicative of the celiac diagnosis, penalized logistic regression with the least absolute shrinkage and selection operator (LASSO) was utilized. When there is a large starting collection of variables, the LASSO model enables the selection of variables with the best predictive capacity. LASSO regression narrowed the beta coefficients of irrelevant variables to zero to select variables. After choosing the set of variables that maximizes the area under the curve (AUC), the value of a penalty parameter (lambda), which controls the amount of shrinkage, is determined using cross validation (10-fold, repeated five times).

Lastly, a ROC curve was used to assess the efficacy of each selected DE transcript to predict the state of celiac. The AUC was calculated to assess the performance of the classification model. 

For all tests, the threshold for statistical significance was set at *p* < 0.05. All analyses were performed with the open-source statistical R software (version 4.2.2., The R Foundation for Statistical Computing). Finally, DE proteins were examined through STRING (https://string-db.org/ (accessed on 25 February 2023)) and Ingenuity Pathway Analysis (IPA, Qiagen, Hilden, Germany) to identify enriched pathways and construct protein-protein interaction networks.

## Figures and Tables

**Table 1:** Patients’ characteristics. Mann–Whitney U test control (CTRL) versus non-celiac wheat sensitivity (NCWS) group; b chi-squared test or Fisher’s exact test, when appropriate. Hp: Helicobacter pylori; TGA-IgA: transglutaminase immunoglobulin A; EMA: endomysial antibodies; ESR: erythrocyte sedimentation rate; CRP: C-reactive protein; Hb: haemoglobin; BMI: body mass index.

Variable	CTRL	Celiac	*p*-Value
Number of subjects	7	5	
Age (years), mean ± SD	56.3 ± 22.8	45.8 ± 19.5	0.432
Gender (M/F)	1/6	0/5	0.583
Marsh classification, n (%)			
1		1 (20%)	
2		0 (0%)	
3a		1 (20%)	
3b		2 (40%)	
3c		1 (20%)	
H. pylori positive, n (%)	4 (57.1%)	1 (20%)	0.247
tTG-IgA, (U/mL), mean ± SD	1.86 ± 1.35	131.2 ± 91.8	0.016
EMA positive	0 (0%)	4 (80%)	0.01
HLA-DQ2/8, n (%)			
absent	5 (71.4%)	0 (0%)	
DQ2	2 (28.6%)	4 (80%)	
DQ8	0 (0%)	1 (20%)	
DQ2/8 (any)	2 (28.6%)	5 (100%)	0.027
ESR (mm/h), mean ± SD	12.6 ± 4.3	20.8 ± 16.5	0.876
CRP (mg/L), mean ± SD	0.41 ± 0.21	0.54 ± 0.34	0.357
Fecal calprotectin (μg/g), mean ± SD	68 ± 35.5	153.2 ± 222.1	0.442
Hb (g/dL), mean ± SD	12.7 ± 0.8	11.6 ± 0.9	0.037
Ferritin (ng/mL), mean ± SD	24.2 ± 20.2	10.1 ± 6.2	0.053
BMI (kg/m2), mean ± SD	25.8 ± 4.0	22.6 ± 3.1	0.149

## Data Availability

Data available upon request.

## Supplementary Materials

The following supporting information can be downloaded at: https://www.mdpi.com/article/10.3390/ijms24097769/s1.
