# Supplementary material for: Gene Expression Profiling in Coeliac Disease Confirmed the Key Role of the Immune System and Revealed a Molecular Overlap with Non-Celiac Gluten Sensitivity
Source: Int J Mol Sci. 2023 Apr 24;24(9):7769. doi: 10.3390/ijms24097769 (PMC10178871; doi:10.3390/ijms24097769)
Supplement: Supplementary file 1 [file ijms-24-07769-s001.zip › Supplementary Captions.pdf]

Figure S1: Network of genes potentially regulated by Eldr.

Table S1: Differentially expressed gene list. In the table are reported the Probe ID, the official gene name, the gene group, the mean expression of controls, the standard deviation of controls (SD), the mean expression of CeD, the standard deviation of CeD, the false discovery rate (FDR) and the direction of changes: upregulated or downregulated genes. Gene group includes antisense (AS), coding gene, long intergenic non-coding RNA (LINC), long non-coding RNA (lnc), genes of uncertain function (LOC), Pseudogenes (Pseudo) and small nucleolar RNAs (SNORA and SNORD). The gene group is empty when completely unknown. Genes recognized by multiple probes are highlighted in pink.

Table S2: Enriched biological process based on Gene Ontologies.

Table S3: Enriched KEGG, Reactome and Wiki pathways.

Table S4: List of significantly activated canonical pathways.

Table S5: List of upstream regulators.

Table S6: List of disease and physiological functions.

Table S7: DE genes shared between CeD and NCGS; Table S8: List of upstream regulators of DE genes shared between CeD and NCGS.
