# Supplementary figures and images for: Gene Expression Profiling in Coeliac Disease Confirmed the Key Role of the Immune System and Revealed a Molecular Overlap with Non-Celiac Gluten Sensitivity
Source: Int J Mol Sci. 2023 Apr 24;24(9):7769. doi: 10.3390/ijms24097769 (PMC10178871; doi:10.3390/ijms24097769)

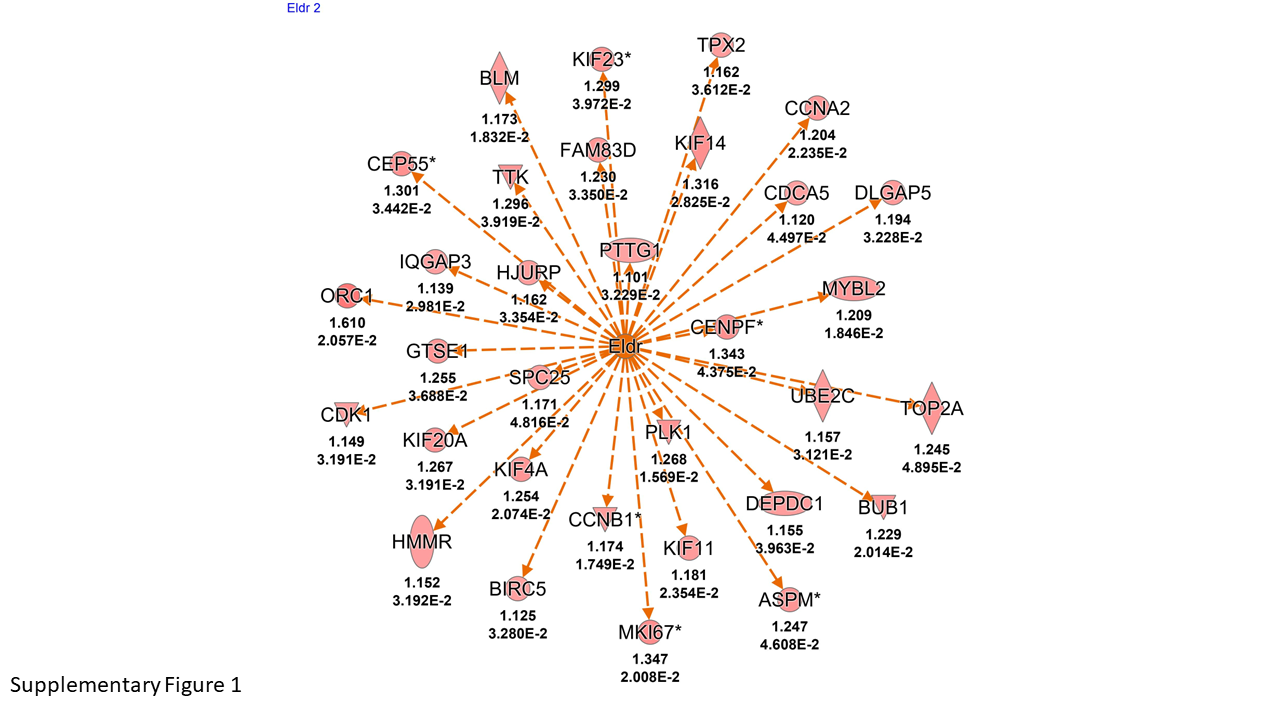

Supplement: Supplementary file 1 [file ijms-24-07769-s001.zip › Supplementary_Figure S1.TIF]
